# Effects of astrocytic mechanisms on neuronal hyperexcitability

**DOI:** 10.1186/1471-2202-15-S1-P221

**Published:** 2014-07-21

**Authors:** Vasily Grigorovsky, Berj L Bardakjian

**Affiliations:** 1 Institute of Biomaterials and Biomedical Engineering, University of Toronto, Toronto, Ontario, M5S 3G9, Canada

## 

Epileptic seizures affect one in hundred people and about 30% of all patients don’t benefit from medication and have to rely on other intervention methods. Current computer models of epilepsy frequently focus on neurons and their interactions to explain both the normal and pathological events in the brain. Recent research has linked glial cell to various cellular processes that are necessary for signal generation and propagation [1]. Several mechanisms by which glial cells could influence the rest of the network have been proposed. One mechanism is the effect of potassium clearance on maintaining homeostatic ion concentration in the extracellular matrix which, when impaired, could lead to seizure-like activity [2]. In addition to directly affecting potassium concentration in the extracellular space (ECS), impaired astrocytes become unable to maintain acceptable intracellular chloride concentrations in the neurons, which can cause or extend seizure-like events [3].

We introduce a computational model of CA3 region of hippocampus, consisting of a network of an astrocyte and a pyramidal cell with a feedback inhibited interneuron, to investigate the effects of a) potassium clearance and buffering by glial cells, and b) calcium propagation on neuronal hyperexcitability. Each cell was represented using modified Traub’s compartment models [4]. Glial contribution was split into two separate factors – K^+^ clearance and buffering, partially revised from Oyehaug [5] and calcium interactions.

**Figure 1:**
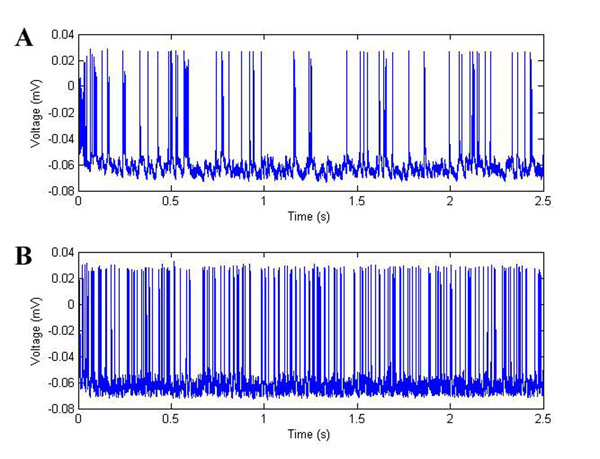
Effects of astrocytic potassium clearance from extracellular space (ECS). (A) Somatic voltage recorded in an interneuron under working potassium clearance. (B) Somatic voltage when clearance mechanisms are disabled and potassium accumulates in the ECS.

We observe that when potassium clearing mechanisms are present (including inwardly rectifying potassium channel as well as phenomenologically represented spatial buffering), neuronal spiking occurs at normal rates (Figure 1A). However when these mechanisms are compromised, potassium accumulation occurs and seizure-like spike trains appear. Preliminary model findings point to both (a) the existence of depolarization block within model parameter searches and (b) the significant influence of glial calcium signaling on spontaneous neuronal discharges.
